# *PNPLA3* and *TM6SF2* genetic variants and hepatic fibrosis and cirrhosis in Pakistani chronic hepatitis C patients: a genetic association study

**DOI:** 10.1186/s12876-022-02469-6

**Published:** 2022-08-26

**Authors:** Bisma Rauff, Badr Alzahrani, Shafiq A. Chudhary, Bilal Nasir, Saqib Mahmood, Munir Ahmad Bhinder, Muhammad Faheem, Ali Amar

**Affiliations:** 1 Storr Liver Centre, Westmead Institute for Medical Research, Westmead Hospital and University of Sydney, Sydney, NSW Australia; 2 Department of Biomedical Engineering, University of Engineering and Technology, Lahore, Narowal Campus, Narowal, Pakistan; 3 Department of Clinical Laboratory Sciences, Jouf University, Sakaka, Kingdom of Saudi Arabia; 4 Institute of Biomedical and Allied Health Sciences, University of Health Sciences, Lahore, Pakistan; 5 Department of Medicine, Lahore General Hospital, Lahore, Pakistan; 6 Department of Human Genetics and Molecular Biology, University of Health Sciences, Lahore, Pakistan; 7 Department of Biological Sciences, National University of Medical Sciences, Rawalpindi, Pakistan

**Keywords:** Genotype–phenotype association, Adiponutrin, *TM6SF2*, Chronic hepatitis C, Pakistan

## Abstract

**Background:**

The present study investigates if common missense functional variants p.I148M and p.E167K in *PNPLA3* and *TM6SF2* genes, respectively, associate with development of hepatic fibrosis and cirrhosis in a geographically novel cohort of Pakistani chronic hepatitis C (CHC) patients.

**Methods:**

In total, 502 Pakistani CHC patients [242 males, median age 40 years, 220 with significant hepatic fibrosis, including 114 with cirrhosis] were genotyped for *PNPLA3* and *TM6SF2* variants using TaqMan genotyping assays. Associations between genotypes, biochemical and clinical parameters were evaluated.

**Results:**

Genotypic distributions for *PNPLA3* and *TM6SF2* polymorphisms conformed to Hardy–Weinberg equilibrium and did not associate with fibrosis grades ≥ F2 or cirrhosis in any of the genetic models tested (all *p* =  > 0.05). *PNPLA3* and *TM6SF2* variants did not modulate baseline characteristics and serum markers of liver injury in CHC patients. Similarly, increasing number of risk alleles of *PNPLA3* and *TM6SF2* polymorphisms had no trend effect on serum liver enzyme activities or proportion of CHC patients with significant or advanced fibrosis or cirrhosis (*p* =  > 0.05). The same trend of no association with hepatic fibrosis or cirrhosis persisted in the multivariate logistic regression models adjusting for age, gender, body mass index and HCV viral load (*p* =  > 0.05).

**Conclusions:**

*PNPLA3* and *TM6SF2* variants do not appear to modulate development of hepatic fibrosis or cirrhosis in present CHC patients of Pakistani origin, and may be of more relevance in liver pathology involving abnormalities in hepatic fat accumulation. These results also reflect the divergent associations observed for different genetic modifiers of hepatic fibrosis and cirrhosis in distinct ethnicities.

**Supplementary Information:**

The online version contains supplementary material available at 10.1186/s12876-022-02469-6.

## Background

Hepatitis C virus (HCV) infection is a major healthcare problem that has chronically infected 58 million people globally. According to WHO estimates, HCV related death toll in 2019 was around 0.29 million, mostly due to hepatic fibrosis and cirrhosis and hepatocellular carcinoma (HCC) [1]. Pakistan has the second largest burden of HCV around the world, with a nationwide prevalence of about 4.8%. Despite the availability of direct-acting antivirals (DAAs) in Pakistan and an associated reduced cost of treatment, the prevalence of HCV still remains persistent, with no indication of decline [2].

Following chronic Hepatitis C (CHC), the natural history of development and progression of chronic liver pathology (CLP) is highly variable and ranges from marginal histological injury to development of liver scarring and eventually HCC. Various factors of viral [e.g. HCV genotype and co-infection with Human Immunodeficiency Virus (HIV)/Hepatitis B Virus (HBV)], metabolic (e.g. metabolic syndrome) and host genetic factors may contribute towards this variability [3]. Indeed, presence of hepatic steatosis and features of metabolic syndrome (including obesity and diabetes mellitus) have been shown to exacerbate progression of hepatic fibrosis and cirrhosis in CHC settings [4].

The dawn of genome-wide association study (GWAS) approach highlighted the potential role of host genetic factors related to hepatic fat metabolism [including PNPLA3 (adiponutrin or patatin-like phospholipase domain-containing protein 3) and TM6SF2 (transmembrane 6 superfamily member 2) in CLP, mainly in non-alcoholic fatty liver disease (NAFLD) settings [5, 6]. In this context, *PNPLA3* rs738409 polymorphism has been found associated with liver fat content [7], as well as with clinical phenotypes of steatosis, steatohepatitis and liver fibrosis/cirrhosis in various hepatic etiologies of non-viral [mainly NAFLD and alcoholic liver disease (ALD)] [8, 9] and viral (mainly CHC) [10, 11] origins. Functionally, carriers of the G allele at the above-mentioned locus have a reduced enzymatic activity of adiponutrin, resulting in high levels of intracellular triglyceride [12], that may subsequently result in the higher predisposition to hepatic scarring and HCC [13].

Similarly, *TM6SF2* rs58542926 variant has been reported to associate with increased risk of NAFLD [14], where the minor (T) allele of this missense variant reportedly conferred TM6SF2 functional loss, translating to enhanced aggregation of intrahepatic lipids due to diminished very low density lipoprotein (VLDL) secretion [15]. TM6SF2 also facilitates the lipidation and secretion of newly formed lipoviro-particles (LVPs) [16]. For that reason, *TM6SF2* rs58542926 variant has been suggested as a potential risk factor for development of liver fibrosis and cirrhosis and linked to lipid abnormalities in chronic HCV patients [16, 17].

To this point, despite the fact that *PNPLA3* and *TM6SF2* polymorphisms represent most commonly investigated lipid metabolism based host genetic variants in NAFLD, their potential influence in CHC mediated CLP is less defined. Additionally, *PNPLA3* and *TM6SF2* variants based genetic predisposition to liver scarring data in CHC patients is mainly available for different Caucasian ethnic groups with scarce representation of other populations, together with Indo-Pak region and mixed populations [18].

In view of the above, the present study explores the association of *PNPLA3* p.I148M (rs738409) and *TM6SF2* p.E167K (rs58542926) single nucleotide polymorphisms (SNPs) with predisposition to liver scarring in a geographically novel cohort (Pakistani CHC patients) employing independent and adjusted analyses for common confounders of demographic, viral, laboratory and clinical nature that may influence this association.

## Methods

### Patients and samples

The details of ethical approvals and consent, patients and samples and their clinical and laboratory evaluations pertaining to this study have been described previously [19]. Briefly, in this cross-sectional comparative study, 502 Pakistani patients with CHC (treatment naive) presenting at a tertiary care hospital in Lahore, Pakistan, were recruited. All patients provided written informed consent for study participation. Ethical guidelines specified in the latest version of Declaration of Helsinki were followed with a priori approval of all study protocols by the Ethical Review Committee for Medical and Biomedical Research, University of Health Sciences, Lahore, Pakistan. CHC was diagnosed considering clinical signs of chronic hepatic ailment along with molecular testing for HCV-RNA. The presence of acute and chronic liver pathologies of non-CHC origin were excluded in all patients. All patients provided venous blood samples for biochemical and genetic analyses and standard demographic, clinical and laboratory evaluation data including liver function tests were recorded for each patient. CHC patients were evaluated for hepatic fibrosis and cirrhosis based on imaging analysis *i.e.* transient elastography by Fibroscan® (Echosens, Waltham, North America) with probes SN77561 and SN94171, where Ziol transient elastography cut-offs [20] were used to define Metavir fibrosis stages (F0–F3) and cirrhosis (F4) with maximal sensitivity and specificity as used previously [19, 21]. Based on the Metavir fibrosis stages, the significant hepatic fibrosis was defined as ≥ F2, advanced hepatic fibrosis as ≥ F3 and hepatic cirrhosis as F4.

### TaqMan assays based molecular analysis of the *PNPLA3* rs738409 and *TM6SF2* rs58542926 SNPs

After isolation of genomic DNA from EDTA anti-coagulated blood samples by means of commercially available DNeasy blood kit (Qiagen, Germany), genotyping of above specified target variants in *PNPLA3* and *TM6SF2* genes was performed using TaqMan genotyping assays (Applied Biosystems, USA) and genotypes were scored using allelic discrimination 7500 Software. All *PNPLA3* and *TM6SF2* molecular analysis was blinded to any phenotypic and clinical information.

### Statistics and expression quantitative trait loci (e‑QTL) analysis

All statistical analyses were undertaken using GraphPad Prism 8.0 and SPSS version 20 for windows unless specified otherwise. Qualitative variables were expressed as frequencies. Whereas, quantitative variables were given as medians with ranges and analyzed using non-parametric Mann–Whitney U or Kruskal–Wallis tests as suitable. Conformance of *PNPLA3* and *TM6SF2* genotypes with Hardy–Weinberg equilibrium (HWE) was analyzed using Chi-square (*χ*^2^) test. Association of *PNPLA3* and *TM6SF2* allelic and genotypic data with phenotypic variables was tested using contingency tables and trend tests. The effects of *PNPLA3* and *TM6SF2* genetic variants as well as any additional risk variables [(including age, sex, body mass index (BMI) and log-transformed HCV-RNA] on liver fibrosis and cirrhosis were determined using univariate and multivariate logistic regression models where the later included any additional risk variables with a *p*-value < 0.20 in univariate analysis, in addition to *PNPLA3* and *TM6SF2* genotypes. Power calculations were performed using the Genetic Association Study (GAS) power calculator available from http://csg.sph.umich.edu/abecasis/gas_power_calculator/index.html. All tests were two-tailed, and *p*-values < 0.05 were considered significant.

GTEx (Genotype-Tissue Expression) dataset version 8 harbors expression quantitative trait loci (eQTL) data for different healthy tissue types (including liver and adipose tissue) and is publicly available at https://www.gtexportal.org/. GTEx was queried with *PNPLA3* p.I148M and TM6SF2 p.E167K SNP identifiers to determine any genotype specific effect of these variants on tissue specific gene expression patterns and hence establish whether these represent loss of function or eQTL variants with respect to their functional significance.

## Results

The baseline data of the present sample set has been described elsewhere [19] and is summarized below. Briefly, among a total of 502 CHC patients, median age was 40 years and there were less men (47.9%) included in the sample set than women. Metavir hepatic fibrosis stage of ≥ F2 was seen in 43.8% CHC patients (including 22.7% cirrhotic cases) (Table 1) and these patients were characterized by significantly higher age, median BMI, HCV-RNA (log_10_), liver enzymes and total bilirubin (all *p* =  < 0.05), but not with respect to gender distribution (*p* =  > 0.05), in comparison with CHC patients presenting with lower grades of hepatic fibrosis (Additional file 1: Supplementary Table 1). Moreover, genotyping efficiency for each of the *PNPLA3* p.I148M and *TM6SF2* p.E167K SNPs was 99.6% (successfully genotyped in 500 individuals) and their genotypic distributions were similar to those reported for Pakistani population from the 1000 genome project (available at http://browser.1000genomes.org) and also did not deviate from Hardy–Weinberg equilibrium (*p* =  > 0.05, *χ*^2^ tests).

**Table 1:** Baseline characteristics, fibrosis stages, genotype and allelic frequencies in the present study sample set

Characteristics	CHC patients (n = 502)
Age (years)	40 (32–50)
Male (n, %)	242 (47.9%)
BMI	26.8 (24–30)
HCV-RNA (log_10_)†	4.7 (3.8–5.5)
ALT (IU/L)	59 (40–87)
AST (IU/L)	56 (39–81)
Total bilirubin (mg/dL)	0.8 (0.7–0.9)
Liver stiffness (kPa)	7.9 (5.5–13.9)
*Metavir stage*	
F0-F1	282 (56.2%)
F2	25 (5%)
F3	81 (16.1%)
F4	114 (22.7%)
*PNPLA3 rs738409 genotypes & alleles (n* = *500)*	
CC	307 (61.4%)
CG	160 (32%)
GG	33 (6.6%)
C	774 (77.4%)
G	226 (22.6%)
*TM6SF2 rs58542926 genotypes & alleles (n* = *500)*	
CC	431 (86.2%)
CT	65 (13%)
TT	04 (0.8%)
C	927 (92.7%)
T	73 (7.3%)

*ALT* alanine transaminase; *AST* aspartate transaminase; *CHC* chronic hepatitis C; *BMI* body mass index

^†^Log-transformed values of HCV-RNA viral load are represented here which were originally estimated in IU/ml units

**Fig. 1:**
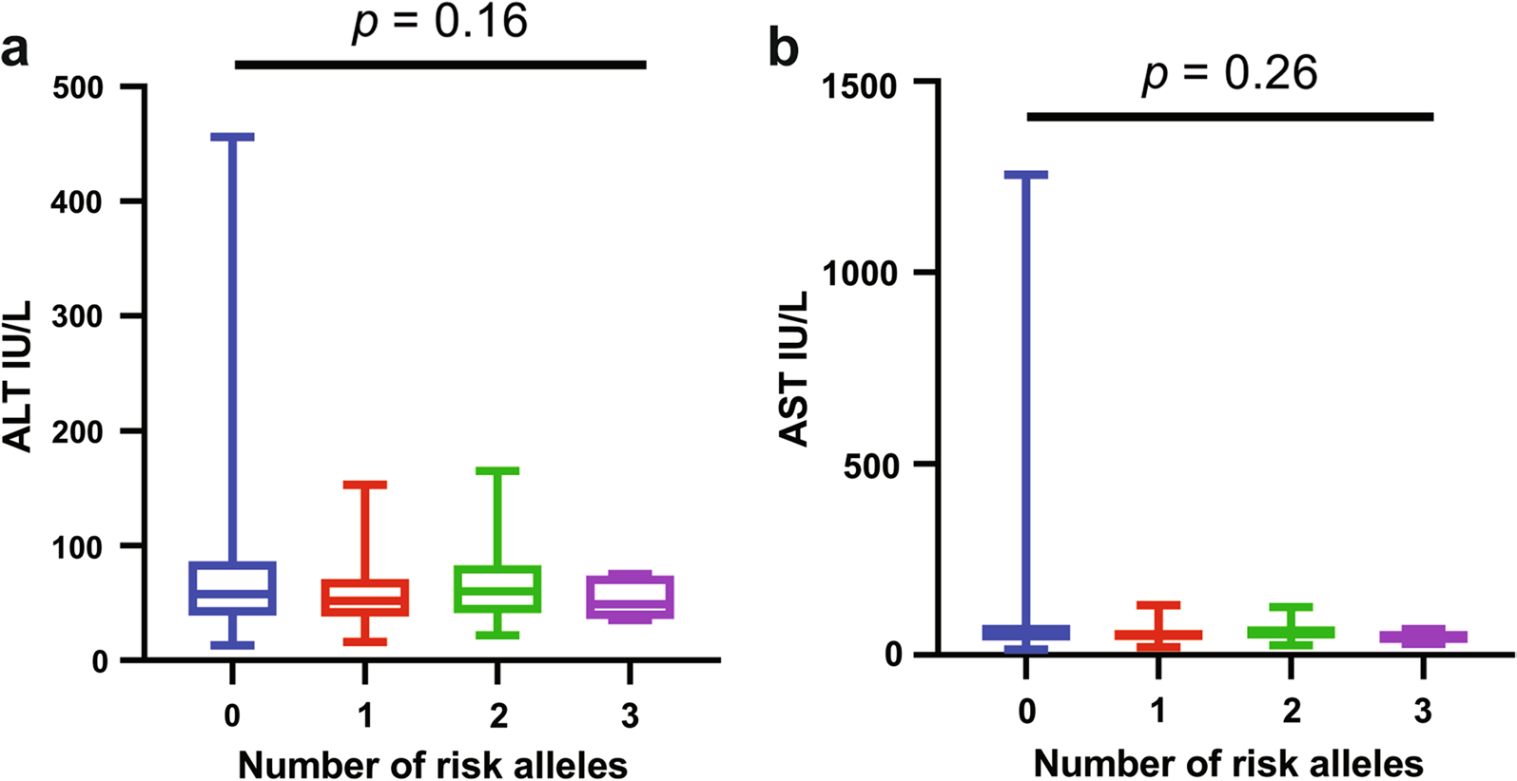
Pooled analysis of the *PNPLA3* rs738409 and *TM6SF2* rs58542926 polymorphisms variant alleles on hepatic enzymes including **a** ALT and **b** AST. The frequencies of the respective risk alleles were as follows; 0 risk alleles = 246, 1 risk allele = 127, 2 risk alleles = 39, and 3 risk alleles = 07

When analyzing association of *PNPLA3* rs738409 and *TM6SF2* rs58542926 polymorphisms with baseline clinical and laboratory characteristics, *PNPLA3* was found to be significantly associated with age (*p* = 0.008). However, no other associations with baseline characteristics, including liver function tests, were apparent for *PNPLA3* and *TM6SF2* SNPs (all *p* =  > 0.05) as presented in Table 2. Also, Fig. 1 demonstrates that no significant increase in serum markers of hepatic injury was evident with an increase in the number of risk alleles of either genetic variant (*p* =  > 0.05).

**Table 2:** Patient baseline characteristics and serum markers of liver injury stratified by the *PNPLA3* rs738409 and *TM6SF2* rs58542926 polymorphisms

Baseline characteristics	*PNPLA3* rs738409 (Recessive model)		*p*-value	*TM6SF2* rs58542926 (Dominant model)		*p*-value
	CC-CG (n = 466)	GG (n = 33)		CC (n = 430)	CT-TT (n = 69)	
Age (years)	40 (33–50)	35 (28–43)	**0.008**	40 (32–50)	40 (32–50)	0.94
Male (n, %)	222 (47.6%)	17 (51.5%)	0.80	205 (47.7%)	34 (49.3%)	0.91
BMI	26.8 (24–30.1)	27 (24–29.2)	0.71	26.8 (23.9–30)	26.9 (24.1–29.9)	0.95
HCV-RNA (log_10_)†	4.66 (3.8–5.5)	4.84 (3.9–4.8)	0.50	4.7 (3.8–5.5)	4.5 (3.5–5.6)	0.47
*Serum markers of liver injury*						
ALT (IU/L)	58 (40–85)	66 (38.2–111.2)	0.51	58 (39–87)	64.5 (42–78)	0.57
AST (IU/L)	56 (39–80)	59.5 (34.5–112.5)	0.68	55 (39–80)	60.5 (40.2–93.2)	0.28
Total bilirubin (mg/dL)	0.8 (0.7–0.9)	0.8 (0.6–0.9)	0.97	0.8 (0.7–0.9)	0.8 (0.7–0.9)	0.99

*ALT* alanine transaminase; *AST* aspartate transaminase; *BMI* body mass index

Statistically significant *p*-values are presented in bold text

^†^Log-transformed values of HCV-RNA viral load are represented here which were originally estimated in IU/ml units

**Fig. 2:**
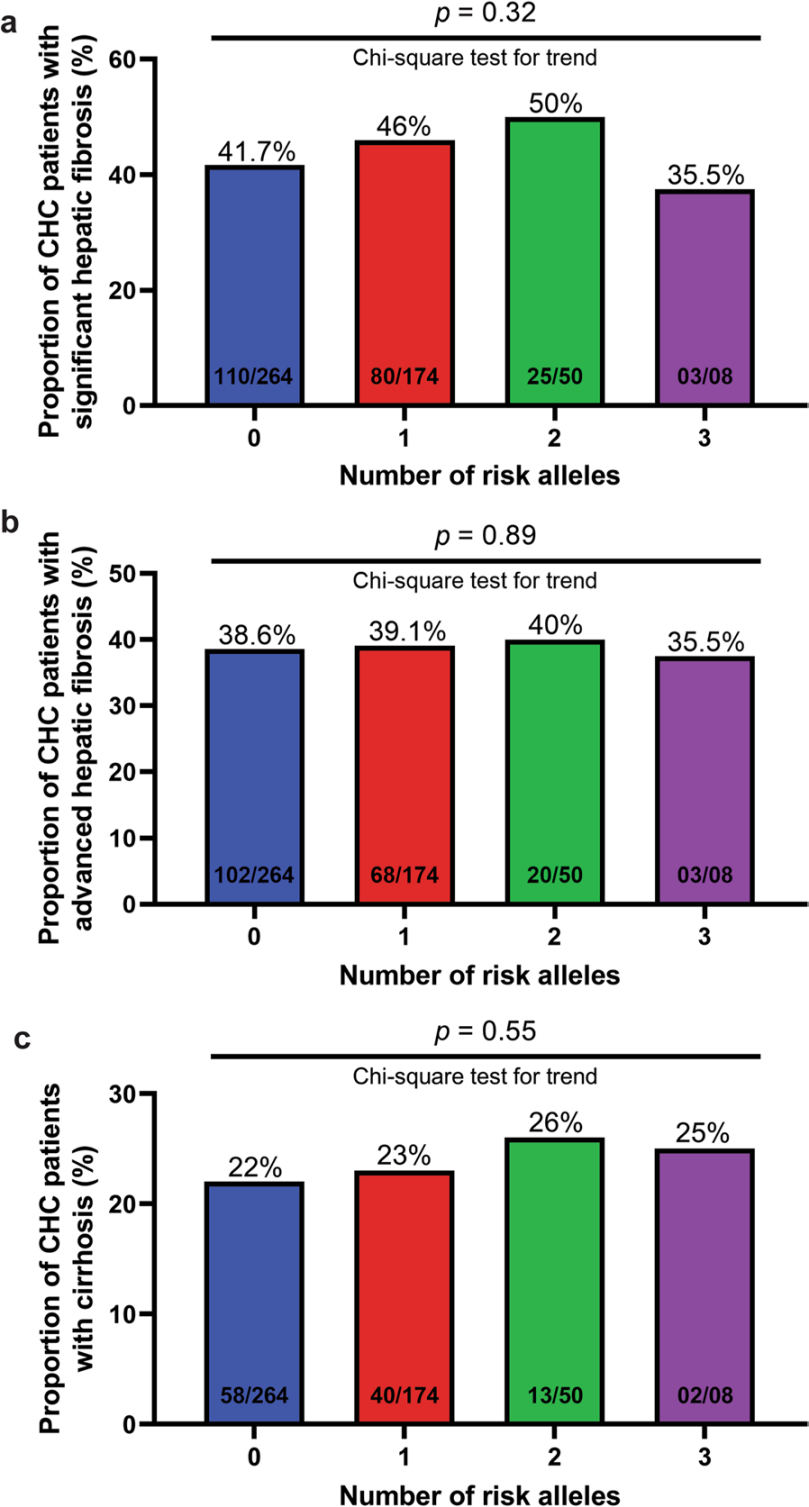
Proportion of **a** significant hepatic fibrosis (≥ F2), **b** advanced hepatic fibrosis (≥ F3), and **c** hepatic cirrhosis (F4) in all CHC patients with 0, 1, 2 and 3 risk alleles of *PNPLA3* and *TM6SF2* variants

Subsequently, we examined any potential role of *PNPLA3* rs738409 and *TM6SF2* rs58542926 variants in moderating predisposition to significant and advanced hepatic fibrosis, and liver cirrhosis in separate analyses. We detected no significant association of *PNPLA3* with liver fibrosis stages F2-F3 and cirrhosis F4 in any of the genetic models tested (*p* =  > 0.05). Likewise, allele and genotype distributions for *TM6SF2* p.E167K missense variant did not differ significantly in CHC patients with or without hepatic fibrosis or cirrhosis even after stratifying the data according to different genetic models (*p* =  > 0.05). These findings are presented in Tables 3 and 4 and Additional file 2: Supplementary Table 2. Considering the risk allele frequency of *PNPLA3* p.I148M variant in the present sample set, these analyses had sufficient power (> 95%) to detect genetic influences on hepatic fibrosis but not on liver cirrhosis (power of study < 50%). The analyses to detect *TM6SF2* genetic association with advanced hepatic fibrosis were powered at > 88%, however, the same for determining predisposition to hepatic cirrhosis were underpowered (< 42%). In addition, no statistically significant increasing trend of hepatic fibrosis or cirrhosis was observable with increase in number of *PNPLA3* and *TM6SF2* risk alleles (Fig. 2).

**Table 3:** Distribution of alleles and genotypes for *PNPLA3* rs738409 and *TM6SF2* rs58542926 polymorphisms and association tests considering significant hepatic fibrosis

*PNPLA3* rs738409 and *TM6SF2* rs58542926 genotypes/alleles	Frequency in CHC patients n (%)	
	Fibrosis grade F0-F1 (n = 278)	Fibrosis grade ≥ F2 (n = 218)
*PNPLA3*		
CC	176 (63.3%)	127 (58.3%)
CG	86 (30.9%)	74 (33.9%)
GG	16 (5.8%)	17 (7.8%)
C	438 (79%)	328 (75%)
G	118 (21%)	108 (25%)
*TM6SF2*		
CC	239 (86%)	189 (86.7%)
CT	37 (13.3%)	27 (12.4%)
TT	02 (0.7%)	02 (0.9%)
C	515 (93%)	405 (93%)
T	41 (07%)	31 (07%)
OR statistics	OR (95% CI)	*p*-value
*PNPLA3*		
CC vs GG (genotypic model)	1.47 (0.72–3.02)	0.45
CC vs CG-GG (dominant model)	1.24 (0.86–1.78)	0.25
CC-CG vs GG (recessive model)	1.38 (0.68–2.81)	0.37
C vs G (allelic model)	1.22 (0.91–1.65)	0.21
*TM6SF2*		
CC vs TT (genotypic model)	1.26 (0.18–9.06)	0.93
CC vs CT-TT (dominant model)	0.94 (0.56–1.58)	0.82
CC-CT vs TT (recessive model)	1.28 (0.18–9.14)	0.81
C vs T (allelic model)	0.96 (0.59–1.56)	1.0

*95% CI* 95% confidence interval; *CHC* chronic hepatitis C; *OR* odds ratio

**Table 4:** Distribution of alleles and genotypes for *PNPLA3* rs738409 and *TM6SF2* rs58542926 genetic variants and association tests with respect to hepatic cirrhosis

*PNPLA3* rs738409 and *TM6SF2* rs58542926 genotypes/alleles	Frequency in CHC patients n (%)	
	Fibrosis grade F0-F3 (n = 383)	Cirrhosis F4 (n = 113)
*PNPLA3*		
CC	234 (61.1%)	69 (61.1%)
CG	124 (32.4%)	36 (31.9%)
GG	25 (6.5%)	08 (7.1%)
C	592 (77%)	174 (77%)
G	174 (23%)	52 (23%)
*TM6SF2*		
CC	334 (87.2%)	94 (83.2%)
CT	46 (12%)	18 (15.9%)
TT	03 (0.8%)	01 (0.9%)
C	714 (93%)	206 (91%)
T	52 (07%)	20 (09%)
OR statistics	OR (95% CI)	*p*-value
*PNPLA3*		
CC vs GG (genotypic model)	1.09 (0.47–2.51)	0.98
CC vs CG-GG (dominant model)	1.00 (0.65–1.54)	0.99
CC-CG vs GG (recessive model)	1.09 (0.48–2.49)	0.84
C vs G (allelic model)	1.02 (0.71–1.45)	1.00
*TM6SF2*		
CC vs TT (genotypic model)	1.18 (0.12–11.52)	0.56
CC vs CT-TT (dominant model)	1.38 (0.77–2.45)	0.28
CC-CT vs TT (recessive model)	1.13 (0.12–10.98)	0.92
C vs T (allelic model)	1.33 (0.78–2.28)	0.36

*95% CI* 95% confidence interval; *CHC* chronic hepatitis C; *OR* odds ratio

Next, potential risk factors that may be associated with liver fibrosis and cirrhosis were analyzed using logistic regression models, as summarized in Table 5 and Additional file 3: Supplementary Table 3, the results of which suggest no significant associations of *PNPLA3* p.I148M and *TM6SF2* p.E167K genotypes with liver fibrosis stages or cirrhosis in univariate and multivariate regression models (all *p* =  > 0.05). Since BMI was independently associated with hepatic fibrosis and cirrhosis in these analyses, we therefore also stratified CHC patients according to BMI status into obese (BMI ≥ 25) and non-obese (BMI < 25) sub-groups and analyzed for any potential genetic associations of *PNPLA3* and *TM6SF2* variants. However, no significant genetic associations were observed for hepatic fibrosis and cirrhosis after stratification according to BMI status (all *p* =  > 0.05), as presented in Additional files 4: Supplementary Fig. 1 and 5: Supplementary Fig. 2.

**Table 5:** Risk factors for the development of significant hepatic fibrosis and cirrhosis in the present CHC cohort

Risk factor	OR (95% CI)	*p*-value
Significant hepatic fibrosis (≥ F2)		
*Univariate regression analysis*		
Age (years)	1.06 (1.04–1.08)	**0.000**
Male (n, %)	1.36 (0.95–1.93)	0.09
BMI	1.05 (1.02–1.09)	**0.005**
HCV-RNA (log_10_)†	1.24 (1.06–1.45)	**0.008**
*PNPLA3* rs738409 (Dominant model)	1.24 (0.86–1.78)	0.25
*TM6SF2* rs58542926 (Recessive model)	1.28 (0.18–9.14)	0.81
*Multivariate regression analysis*		
Age (years)	1.06 (1.04–1.08)	**0.000**
Male (n, %)	1.54 (1.04–2.28)	**0.033**
BMI	1.06 (1.02–1.10)	**0.006**
HCV-RNA (log_10_)^a^	1.20 (1.01–1.42)	**0.040**
*PNPLA3* rs738409 (Dominant model)	1.39 (0.94–2.07)	0.10
*TM6SF2* rs58542926 (Recessive model)	1.38 (0.16–11.80)	0.09
Hepatic cirrhosis (F4)		
*Univariate regression analysis*		
Age (years)	1.05 (1.03–1.07)	**0.000**
Male (n, %)	1.39 (0.91–2.12)	0.12
BMI	1.04 (1.00–1.09)	**0.036**
HCV-RNA (log_10_)†	1.06 (0.89–1.28)	0.51
*PNPLA3* rs738409 (Recessive model)	1.09 (0.48–2.50)	0.84
*TM6SF2* rs58542926 (Dominant model)	1.38 (0.77–2.45)	0.28
*Multivariate regression analysis*		
Age (years)	1.05 (1.03–1.07)	**0.000**
Male (n, %)	1.58 (1.01–2.48)	**0.046**
BMI	1.05 (1.01–1.10)	**0.028**
*PNPLA3* rs738409 (Recessive model)	1.48 (0.62–3.54)	0.38
*TM6SF2* rs58542926 (Dominant model)	1.43 (0.78–2.63)	0.25

*95% CI* 95% confidence interval; *BMI* body mass index; *CHC* chronic hepatitis C; *OR* odds ratio

Statistically significant *p*-values are presented in bold text

^†^Log-transformed values of HCV-RNA viral load are represented here which were originally estimated in IU/ml units

We further explored genotype-gene expression based functional significance of analyzed genetic variants using GTEx database (Additional files 6: Supplementary Table 4 and 7: Supplementary Table 5) and found that PNPLA3*rs738409 and TM6SF2*rs58542926 do not represent significant eQTLs/sQTLs of *PNPLA3* and *TM6SF2* gene expression in liver and visceral adipose tissues, but may modulate genotype specific target gene expression in other human peripheral tissues (of note in subcutaneous adipose tissue for TM6SF2*rs58542926 eQTL pair) as presented in Additional files 8: Supplementary Fig. 3 and 9: Supplementary Fig. 4.

## Discussion

*PNPLA3* rs738409 and *TM6SF2* rs58542926 SNPs, that initially came into highlight from GWAS [5] and EWAS [6] analyses in patients with NAFLD, are important modulators of hepatic fat metabolism and are regarded as significant genetic influencers of liver injury of various etiologies especially steatosis and NAFLD. The current study, which examined these variants in the setting of Pakistani CHC patients, suggests that *PNPLA3* and *TM6SF2* polymorphisms seem to have no effect on serum markers of hepatic injury nor they associate with development of hepatic scarring following CHC infection.

From a mechanistic perspective, wild-type PNPLA3 has a marked hepatic expression where it exhibits triglyceride lipolytic activity in hepatocytes as well as retinyle-palmitate lipase activity in stellate cells of liver [22]. *PNPLA3* p.I148M represents a loss of function variant resulting in reduced lipase activity and gain of hepatic lipogenic activity manifesting as hepatic fat accumulation [23]. It may further promote liver injury by influencing retinol remodeling [24] and promoting production of pro-fibrotic proteins in stellate cells of liver [25]. In a similar way, wild-type TM6SF2 activity mediates Apolipoprotein B (APOB) lipidation and hepatic efflux of triglycerides to circulation through VLDLs [26]. *TM6SF2* p.E167K variant affects modulation of triglyceride-rich lipoproteins and APOB thereby increasing hepatic fat content [6, 27]. Altogether, *PNPLA3* p.I148M and *TM6SF2* p.E167K variants may promote steatosis and steatohepatitis which may indirectly predispose to progression of liver scarring (fibrosis and cirrhosis).

Indeed, the association of *PNPLA3* rs738409 and *TM6SF2* rs58542926 SNPs with hepatic injury resulting from dysregulation of hepatic lipid metabolism (steatohepatitis and NAFLD) is well established [5, 6, 8, 17, 28], however, extension of their implication in NAFLD-associated hepatic fibrosis is not definite [28] and is certainly debatable in the context of hepatic fibrosis and cirrhosis of viral (especially CHC) etiology [10, 17, 29, 30].

Previously, several studies based on primarily Caucasian and some Asian datasets have suggested association of *PNPLA3* risk variant with increased severity of hepatic fibrosis and cirrhosis [30, 31] and enhanced progression of liver fibrosis or cirrhosis [29] or independent steatosis [10, 32] in CHC settings. However, such evidence is relatively limited for *TM6SF2* where only a couple of studies reported overrepresentation of *TM6SF2* rs58542926 risk allele in CHC patients displaying hepatic scarring (fibrosis and cirrhosis) [30, 33]. Extension of *PNPLA3* and *TM6SF2* association has also been observed to hepatocellular carcinoma (HCC) [34] attributable to both viral [13] and non-viral [35, 36] origin, and less commonly to alcoholic liver disease [9]. In addition, *PNPLA3* and *TM6SF2* variants have been regarded as variants of interest for risk of liver steatosis even in transplant settings [37, 38]. However, potential association of *PNPLA3* and *TM6SF2* genetic polymorphisms with HCC is not without several contradictions [9, 39–41].

Nevertheless, in the present CHC patients of Pakistani origin we did not detect any significant effect of *PNPLA3* and *TM6SF2* variants in modulating serum markers of hepatic injury nor hepatic fibrosis and cirrhosis. The outcomes of our study are in line with two major such studies from Caucasian and Asian populations. Eslam et al. suggested only a marginal association if any (that was not persistent after adjustment for confounders) of *TM6SF2* with fibrosis severity and none for fibrosis progression in a large sample set of CHC patients (n = 2023) from the International Liver Disease Genetics Consortium [17]. Likewise, Huang and colleagues demonstrated a role of *PNPLA3* variant in HCV induced steatosis but no independent association with liver fibrosis and cirrhosis based on 1080 CHC patients of Chinese ancestry [11]. The same pattern (association of *PNPLA3* with steatosis and steatohepatitis but not with hepatic scarring in CHC patients) was also evident from another Caucasian study [10]. Also, no significant impact of *PNPLA3* and *TM6SF2* variants on hepatic fibrosis and cirrhosis of CHC background was highlighted in some of such studies from other populations [14, 42, 43]. It is noteworthy here that all these studies had biopsy proven staging and diagnoses of hepatic fibrosis and cirrhosis. Interestingly, a couple of studies reported reduced HCV viral load associated with *PNPLA3* [29] and *TM6SF2* [17] risk alleles, translating to reduced HCV lipidation and infectivity [16], however, no such associations were observed in our study.

Only a single study on the present subject is available from Pakistan that reported a significant prevalence of *PNPLA3* risk variant, but not *TM6SF2* polymorphism, in end stage hepatic ailment patients with mixed etiology (viral as well as non-viral origin) who received living donor liver transplantation, when compared to normal controls [18].

The reasons for discrepancies observed in the association of *PNPLA3* and *TM6SF2* variants in the CHC context, including this very study, are not certain but few possibilities can be entertained. Differences in ways to evaluate hepatic fibrosis and cirrhosis *i.e.* liver biopsy as the gold standard vs use of imaging techniques (such as transient elastography or Fibroscan), and additional variation in elastography thresholds used to define different stages of liver fibrosis and cirrhosis in different clinical settings, may have a part to play. In addition, most studies lack concomitant assessment of hepatosteatosis in CHC settings and functional correlation with lipid profile since these may modulate susceptibility to liver fibrosis and cirrhosis as highlighted in previous studies [10, 11, 17]. Further, study characteristics may display inherent differences especially concerning patient selection (treatment naïve vs those who received prior anti-viral treatment), baseline characteristics (varied prevalence of different HCV genotypes), sample sizes and resultant study powers and data analysis approaches, which may also offer to explain a part of the heterogeneity in these genetic predisposition results. Lastly, population to population dissimilarities in risk allele frequencies for *PNPLA3* & *TM6SF2* SNPs in general population of Europeans (MAF of 22.6% and 6.8% for *PNPLA3* and *TM6SF2*, respectively) vs Chinese (MAF of 38.3% and 4.4% for *PNPLA3* and *TM6SF2*, respectively) vs Pakistani (MAF of 19.8% and 8.3% for *PNPLA3* and *TM6SF2*, respectively) may also have an effect in this regard.

Data regarding genotype and tissue specific gene expression patterns for *PNPLA3* p.I148M and *TM6SF2* p.E167K variants and their functional significance are limited [10, 17, 44, 45]. Therefore, we used the modern GTEx database to determine influence of *PNPLA3* p.I148M and *TM6SF2* p.E167K variants on their respective gene expression, however, no genotype specific modulation of hepatic or visceral adipose tissue gene expression of *PNPLA3* and TMS6F2 was evident. This finding is consistent with gene expression studies on NAFLD and CHC patient samples [10, 44], but contrary to a couple of available reports [17, 45]. These data largely emphasizes and support that *PNPLA3* and *TM6SF2* missense variants may effect function and/or structure of their coded proteins rather than gene expression [6, 23, 27]. This functional mechanistic clarification may be of relevance in designing any therapeutic manipulation of *PNPLA3* and *TM6SF2* in liver pathologies in future.

This study is not without some limitations. First, absence of metabolic and serum lipid profiles and assessments of steatosis and steatohepatitis data considering lipid metabolism related effects of *PNPLA3* and especially *TM6SF2* variants analyzed is a limitation, which would have otherwise provided useful insights to the study in this regard. Second, diabetes mellitus status (a known significant risk factor for the development of liver cirrhosis in CHC patients), including any Homeostatic Model Assessment for Insulin Resistance (HOMA-IR) data, was not available and hence, regression models analyzing association of *PNPLA3* and *TM6SF2* variants were not adjusted for any potential effects of diabetes mellitus status, which adds to the limitations of this study. Third, though we tried to use a sizeable sample set of 502 CHC patients for the present study, the sample sizes in the stratified analyses were rather small and may have rendered association tests for *TM6SF2* variant underpowered as suggested by power of study calculations.

## Conclusions

In conclusion, *PNPLA3* and *TM6SF2* genetic polymorphisms do not appear as major determinants of liver scarring in Pakistani patients with CHC and may be of more relevance to CLP with dysregulated hepatic fat such as NAFLD. Further studies with large sample sizes, simultaneous assessment of metabolic and lipid related histological profiles and having functional analyses support in diverse ethnicities may elucidate potential role and clinical utility of *PNPLA3* and *TM6SF2* genetic testing in CHC mediated liver scarring.

## Supplementary Information

**Additional file 1. Supplementary Table 1.** Baseline characteristics according to fibrosis (Metavir) stages.

**Additional file 2. Supplementary Table 2.** Distribution of alleles and genotypes for *PNPLA3* rs738409 and *TM6SF2* rs58542926 polymorphisms and association tests with respect to advanced hepatic fibrosis.

**Additional file 3. Supplementary Table 3.** Risk factors for the development of advanced hepatic fibrosis in the present CHC cohort.

**Additional file 4. Supplementary Fig. 1.** Genetic association analyses of *PNPLA3* variant (recessive model) with (a) significant hepatic fibrosis (≥ F2), **(b)** advanced hepatic fibrosis (≥ F3), and **(c)** hepatic cirrhosis (F4) after stratification of CHC patients into obese and non-obese groups based on BMI status.

**Additional file 5. Supplementary Fig. 2.** Genetic association analyses of *TM6SF2* variant (dominant model) with (a) significant hepatic fibrosis (≥ F2), (b) advanced hepatic fibrosis (≥ F3), and (c) hepatic cirrhosis (F4) after stratification of CHC patients into obese and non-obese groups based on BMI status.

**Additional file 6. Supplementary Table 4.** eQTL and sQTL analysis for PNPLA3*rs738409 using GTEx database.

**Additional file 7. Supplementary Table 5.** eQTL analysis for TM6SF2*rs58542926 using GTEx database.

**Additional file 8. Supplementary Fig. 3.** Multi-tissue eQTL comparison for PNPLA3*rs738409 using GTEx dataset.

**Additional file 9. Supplementary Fig. 4.** Multi-tissue eQTL comparison for TM6SF2*rs58542926 using GTEx dataset.

**Additional file 10.** Raw sample set data for Pakistani CHC patients.

## Data Availability

All data generated or analyzed during this study are included in this published article (and its supplementary information files including Additional file 10 for raw sample set data).

## Abbreviations

ALD: Alcoholic liver disease
APOB: Apolipoprotein B
BMI: Body mass index
CHC: Chronic hepatitis C
CLP: Chronic liver pathology
DAAs: Direct-acting antivirals
EDTA: Ethylenediaminetetraacetic acid
eQTL: Expression quantitative trait loci
GAS: Genetic association study
GTEx: Genotype-tissue expression
GWAS: Genome-wide association study
HBV: Hepatitis B Virus
HCC: Hepatocellular carcinoma
HCV: Hepatitis C virus
HIV: Human immunodeficiency virus
HOMA-IR: Homeostatic model assessment for insulin resistance
HWE: Hardy–Weinberg equilibrium
LVPs: Lipoviro-particles
MAF: Minor allele frequency
NAFLD: Non-alcoholic fatty liver disease
PNPLA3: Patatin-like phospholipase domain-containing protein 3
SNPs: Single nucleotide polymorphisms
TM6SF2: Transmembrane 6 superfamily member 2
VLDL: Very low density lipoprotein
