# Supplementary material for: PNPLA3 and TM6SF2 genetic variants and hepatic fibrosis and cirrhosis in Pakistani chronic hepatitis C patients: a genetic association study
Source: BMC Gastroenterol. 2022 Aug 26;22:401. doi: 10.1186/s12876-022-02469-6 (PMC9414345; doi:10.1186/s12876-022-02469-6)
Supplement: Supplementary file 1 — Additional file 1. Supplementary Table 1. Baseline characteristics according to fibrosis (Metavir) stages. [file 12876_2022_2469_MOESM1_ESM.docx]

**Supplementary Table 1.** Baseline characteristics according to fibrosis (Metavir) stages.

| **Characteristics** | **Metavir stage F0-F1 (n = 282)** | **Metavir stage F2 (n = 25)** | **Metavir stage F3 (n = 81)** | **Metavir stage F4 (n = 114)** | **Overall *p*- value** | **Significant multiple comparison *p*-values^a,c^** |
| --- | --- | --- | --- | --- | --- | --- |
| Age (years) | 36 (30-45) | 40 (36-49) | 42 (35-50) | 45 (38-54) | **<0.0001^a^** | F0-F1 vs F3 = **<0.0001**  F0-F1 vs F4 = **<0.0001** |
| Male (n, %) | 156 (55.3%) | 15 (60%) | 38 (46.9%) | 52 (45.6%) | 0.20**^b^** | - |
| BMI | 26.2 (23.6-29.5) | 29.5 (24.8-33.2) | 26.7 (23.8-30.9) | 27.3 (24.9-30.9) | **0.018^a^** | F0-F1 vs F4 = **0.046** |
| HCV-RNA (log_10_)† | 4.4 (3.7-5.4) | 5 (4.5-5.6) | 4.9 (4.1-5.8) | 4.8 (3.7-5.7) | **0.032^a^** | ns |
| ALT (IU/L) | 54 (37-74.8) | 61 (47.8-101.3) | 59 (38.8-116.8) | 74 (55-120.5) | **<0.0001^a^** | F0-F1 vs F4 = **<0.0001** |
| AST (IU/L) | 49.5 (35-68) | 62.5 (41.8-93.5) | 65 (37.5-98) | 69.5 (49.2-120) | **<0.0001^a^** | F0-F1 vs F3 = **0.017**  F0-F1 vs F4 = **<0.0001** |
| Total bilirubin (mg/dL) | 0.8 (0.6-0.9) | 0.8 (0.7-0.9) | 0.8 (0.7-0.9) | 0.8 (0.7-1) | **0.008^a^** | F0-F1 vs F4 = **0.004** |

ALT, alanine transaminase; AST, aspartate transaminase; BMI, body mass index; ns, not significant. Statistically significant *p*-values are presented in bold text.

†Log-transformed values of HCV-RNA viral load are represented here which were originally estimated in IU/ml units.

**^a^***p*-value from non-parametric Kruskal-Wallis test.

**^b^***p*-value from Chi-square test.

**^c^**Sub-group specific significant multiple comparison *p*-values (from Dunn’s multiple comparison test) that derive the overall significant *p*-value for each variable compared across different Metavir stages.
