# Supplementary material for: PNPLA3 and TM6SF2 genetic variants and hepatic fibrosis and cirrhosis in Pakistani chronic hepatitis C patients: a genetic association study
Source: BMC Gastroenterol. 2022 Aug 26;22:401. doi: 10.1186/s12876-022-02469-6 (PMC9414345; doi:10.1186/s12876-022-02469-6)
Supplement: Supplementary file 2 — Additional file 2. Supplementary Table 2. Distribution of alleles and genotypes for PNPLA3 rs738409 and TM6SF2 rs58542926 polymorphisms and association tests with respect to advanced hepatic fibrosis. [file 12876_2022_2469_MOESM2_ESM.docx]

**Supplementary Table 2.** Distribution of alleles and genotypes for *PNPLA3* rs738409 and *TM6SF2* rs58542926 polymorphisms and association tests with respect to advanced hepatic fibrosis.

| ***PNPLA3* rs738409 and *TM6SF2* rs58542926 genotypes/alleles** | **Frequency in CHC patients n (%)** | |
| --- | --- | --- |
|  | **Fibrosis grade F0-F2**  **(n = 303)** | **Fibrosis grade ≥F3**  **(n = 193)** |
| ***PNPLA3*** | | |
| CC | 187 (61.7%) | 116 (60.1%) |
| CG | 98 (32.3%) | 62 (32.1%) |
| GG | 18 (5.9%) | 15 (7.8%) |
| C | 472 (78%) | 294 (76%) |
| G | 134 (22%) | 92 (24%) |
| ***TM6SF2*** | | |
| CC | 258 (85.2%) | 170 (88.1%) |
| CT | 43 (14.2%) | 21 (10.9%) |
| TT | 02 (0.7%) | 02 (1.0%) |
| C | 559 (92%) | 361 (94%) |
| T | 47 (08%) | 25 (06%) |
| **OR statistics** | **OR (95% CI)** | ***p*-value** |
| ***PNPLA3*** | | |
| CC vs GG (genotypic model) | 1.34 (0.65-2.77) | 0.73 |
| CC vs CG-GG (dominant model) | 1.07 (0.74-1.55) | 0.72 |
| CC-CG vs GG (recessive model) | 1.33 (0.66-2.71) | 0.43 |
| C vs G (allelic model) | 1.10 (0.81-1.49) | 0.58 |
| ***TM6SF2*** | | |
| CC vs TT (genotypic model) | 1.52 (0.21-10.88) | 0.51 |
| CC vs CT-TT (dominant model) | 0.78 (0.45-1.33) | 0.35 |
| CC-CT vs TT (recessive model) | 1.58 (0.22-11.28) | 0.65 |
| C vs T (allelic model) | 0.82 (0.50-1.36) | 0.53 |

95% CI, 95% confidence interval; CHC, chronic hepatitis C; OR, odds ratio
