# Supplementary material for: PNPLA3 and TM6SF2 genetic variants and hepatic fibrosis and cirrhosis in Pakistani chronic hepatitis C patients: a genetic association study
Source: BMC Gastroenterol. 2022 Aug 26;22:401. doi: 10.1186/s12876-022-02469-6 (PMC9414345; doi:10.1186/s12876-022-02469-6)
Supplement: Supplementary file 3 — Additional file 3. Supplementary Table 3. Risk factors for the development of advanced hepatic fibrosis in the present CHC cohort. [file 12876_2022_2469_MOESM3_ESM.docx]

**Supplementary Table 3.** Risk factors for the development of advanced hepatic fibrosis in the present CHC cohort.

| **Risk factor** | **OR (95% CI)** | ***p*-value** |
| --- | --- | --- |
| **Univariate regression analysis** | | |
| Age (years) | 1.06 (1.04-1.07) | **0.000** |
| Male (n, %) | 1.47 (1.02-2.10) | **0.037** |
| BMI | 1.04 (1.00-1.08) | **0.032** |
| HCV-RNA (log_10_)† | 1.18 (1.01-1.38) | **0.040** |
| *PNPLA3* rs738409 (Recessive model) | 1.33 (0.66-2.72) | 0.43 |
| *TM6SF2* rs58542926 (Dominant model) | 0.78 (0.45-1.33) | 0.35 |
| **Multivariate regression analysis** | | |
| Age (years) | 1.06 (1.04-1.08) | **0.000** |
| Male (n, %) | 1.60 (1.08-2.37) | **0.020** |
| BMI | 1.04 (1.00-1.08) | **0.040** |
| HCV-RNA (log_10_)^a^ | 1.12 (0.95-1.33) | 0.19 |
| *PNPLA3* rs738409 (Recessive model) | 1.98 (0.92-4.27) | 0.08 |
| *TM6SF2* rs58542926 (Dominant model) | 0.73 (0.41-1.31) | 0.29 |

95% CI, 95% confidence interval; BMI, body mass index; CHC, chronic hepatitis C; OR, odds ratio. Statistically significant *p*-values are presented in bold text.

†Log-transformed values of HCV-RNA viral load are represented here which were originally estimated in IU/ml units.
