# Supplementary material for: PNPLA3 and TM6SF2 genetic variants and hepatic fibrosis and cirrhosis in Pakistani chronic hepatitis C patients: a genetic association study
Source: BMC Gastroenterol. 2022 Aug 26;22:401. doi: 10.1186/s12876-022-02469-6 (PMC9414345; doi:10.1186/s12876-022-02469-6)
Supplement: Supplementary file 4 — Additional file 4. Supplementary Fig. 1. Genetic association analyses of PNPLA3 variant (recessive model) with (a) significant hepatic fibrosis (≥ F2), (b) advanced hepatic fibrosis (≥ F3), and (c) hepatic cirrhosis (F4) after stratification of CHC patients into obese and non-obese groups based on BMI status. [file 12876_2022_2469_MOESM4_ESM.docx]

**
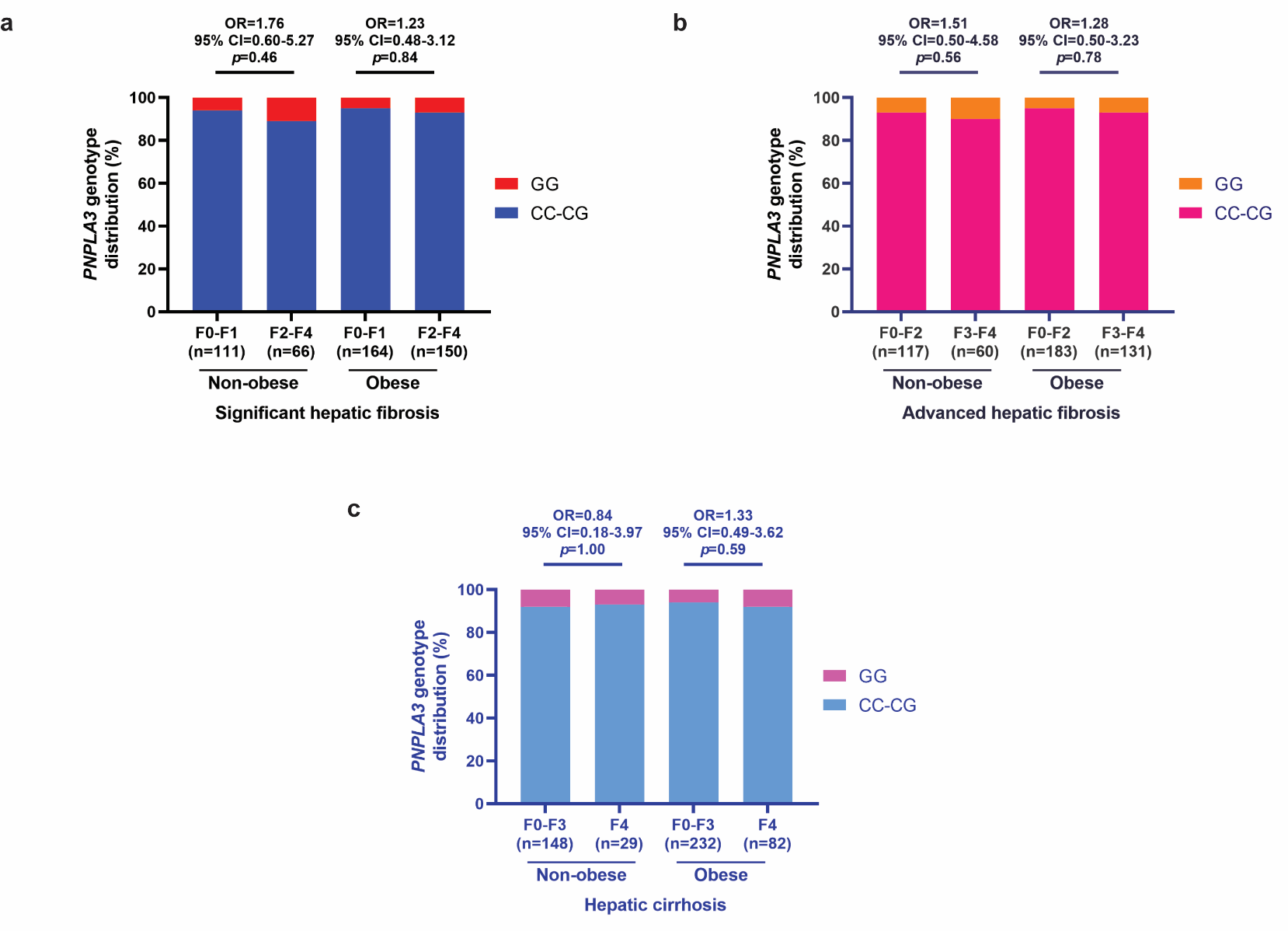
**

**Supplementary Fig. 1.** Genetic association analyses of *PNPLA3* variant (recessive model) with **(a)** significant hepatic fibrosis (≥F2), **(b)** advanced hepatic fibrosis (≥F3), and **(c)** hepatic cirrhosis (F4) after stratification of CHC patients into obese and non-obese groups based on BMI status.
