# Supplementary material for: PNPLA3 and TM6SF2 genetic variants and hepatic fibrosis and cirrhosis in Pakistani chronic hepatitis C patients: a genetic association study
Source: BMC Gastroenterol. 2022 Aug 26;22:401. doi: 10.1186/s12876-022-02469-6 (PMC9414345; doi:10.1186/s12876-022-02469-6)
Supplement: Supplementary file 7 — Additional file 7. Supplementary Table 5. eQTL analysis for TM6SF2*rs58542926 using GTEx database. [file 12876_2022_2469_MOESM7_ESM.docx]

**Supplementary Table 5.** eQTL analysis for *TM6SF2**rs58542926 using GTEx database.

| **Gene** | **Variant ID** | **Ref_Alt** | **Tissue** | **Total samples** | **Genotype** | | | **NES** | ***p*-value** |
| --- | --- | --- | --- | --- | --- | --- | --- | --- | --- |
|  |  |  |  |  | **CC** | **CT** | **TT** |  |  |
| *TM6SF2* (ENSG00000213996.12) | rs58542926 (chr19_19268740_C_T_b38) | C_T | **Nerve - Tibial** | 532 | 462 | 66 | 04 | **0.39** | **2.5 x 10^-5^** |
|  |  |  | **Heart - Left Ventricle** | 386 | 324 | 60 | 02 | **0.39** | **3.2 x 10^-5^** |
|  |  |  | **Adipose - Subcutaneous** | 581 | 502 | 75 | 04 | **0.32** | **7.1 x 10^-5^** |
|  |  |  | **Skin - Sun Exposed (Lower leg)** | 605 | 520 | 81 | 04 | **0.33** | **7.6 x 10^-5^** |
|  |  |  | **Cells - Cultured fibroblasts** | 483 | 427 | 53 | 03 | **0.26** | **2.7 x 10^-4^** |
|  |  |  | Liver | 208 | 183 | 23 | 02 | -0.218 | 2.9 x 10^-3^ |
|  |  |  | Adipose - Visceral (Omentum) | 469 | 402 | 66 | 01 | 0.186 | 0.04 |
|  |  |  | Whole Blood | 670 | NA | | | | |

Alt, alternative allele; Ref, reference allele; NA, not available; and NES, normalized effect size.

Significant e/sQTL-tissue pairs are presented in bold.

Total samples: represents the number of RNA-seq samples for which genotype data is available, NES: defined as slope of the linear regression representing eQTL effect sizes and is determined as the effect of the alternative allele (Alt) relative to the reference allele (Ref) in the human genome reference GRCh38/hg38, *p*-value: from a t-test that compares observed NES from single-tissue eQTL analysis to a null NES of 0.
