# Supplementary figures and images for: PNPLA3 and TM6SF2 genetic variants and hepatic fibrosis and cirrhosis in Pakistani chronic hepatitis C patients: a genetic association study
Source: BMC Gastroenterol. 2022 Aug 26;22:401. doi: 10.1186/s12876-022-02469-6 (PMC9414345; doi:10.1186/s12876-022-02469-6)

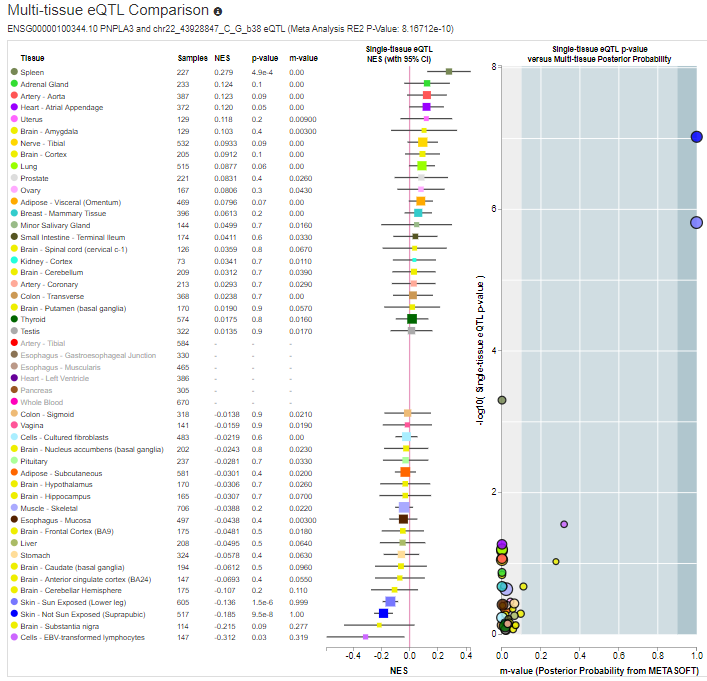


**Supplementary Fig. 3.** Multi-tissue eQTL comparison for *PNPLA3**rs738409 using GTEx dataset.

Supplement: Supplementary file 8 — Additional file 8. Supplementary Fig. 3. Multi-tissue eQTL comparison for PNPLA3*rs738409 using GTEx dataset. [file 12876_2022_2469_MOESM8_ESM.docx]

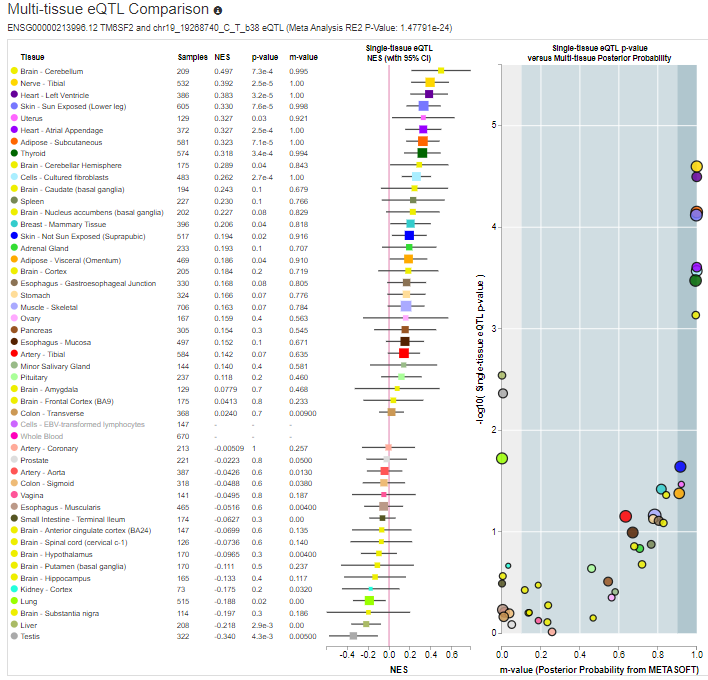


**Supplementary Fig. 4.** Multi-tissue eQTL comparison for *TM6SF2**rs58542926 using GTEx dataset.

Supplement: Supplementary file 9 — Additional file 9. Supplementary Fig. 4. Multi-tissue eQTL comparison for TM6SF2*rs58542926 using GTEx dataset. [file 12876_2022_2469_MOESM9_ESM.docx]
